# The association between prenatal exposure to polycyclic aromatic hydrocarbons and birth weight: A meta-analysis

**DOI:** 10.1371/journal.pone.0236708

**Published:** 2020-08-13

**Authors:** Liren Yang, Li Shang, Shanshan Wang, Wenfang Yang, Liyan Huang, Cuifang Qi, Anil Gurcan, Zixuan Yang, Mei Chun Chung

**Affiliations:** 1 Department of Obstetrics and Gynecology, Maternal & Child Health Center, The First Affiliated Hospital of Xi'an Jiaotong University, Xi’an, Shaanxi, P.R. China; 2 School of Public Health, Xi’an Jiaotong University Health Science Center, Xi’an, Shaanxi, P.R. China; 3 Department of Public Health and Community Medicine, Tufts University School of Medicine, Boston, Massachusetts, United States of America; 4 Antai College, Shanghai Jiao Tong University, Shanghai, P.R. China; National Sun Yat-sen University, TAIWAN

## Abstract

**Background:**

Polycyclic aromatic hydrocarbons (PAHs) are a kind of endocrine disruptors, which can enter human body by the inhalation of PAH-containing matter and the ingestion of PAH-containing foodstuffs. Studies showed that PAHs can cross the placental barrier and might cause adverse effects on the fetus.

**Objectives:**

This meta-analysis aimed to estimate the associations between prenatal exposure to PAHs and birth weight.

**Methods:**

Articles published in English until May 8, 2020 and reported the effects of prenatal exposure to PAHs on birth weight were searched in multiple electronic databases including PubMed, the Web of Science, EMBASE and the Cochrane Library. The included studies were divided into three groups in accordance with the measurement of PAHs exposure. Then coefficient was extracted, conversed and synthesized by random-effects meta-analysis. And risk of bias was assessed for each study.

**Results:**

A total of 3488 citations were searched and only 11 studies were included finally after double assessment. We found that there were no association between PAH-DNA adducts in cord blood (low/high) (OR: 1.0, 95%CI: 0.97, 1.03), 1-hydroxy pyrene (1-HP) concentration in maternal urine (OR: 1.0, 95%CI: 0.97, 1.03) and prenatal maternal airborne PAHs exposure (OR: 0.97, 95%CI: 0.93, 1.01) and birth weight. However, we observed ethnicity may change the effects of PAHs exposure on birth weight.

**Conclusions:**

There is no significant relationship between prenatal exposure to PAHs and birth weight in our meta-analysis. Further studies are still needed for determining the effects of prenatal PAHs exposure on birth weight.

## Introduction

Growing number of studies showed that air pollution might play an important role in the occurrence of adverse pregnancy outcomes [1–3]. A number of meta-analyses conducted in recent years have also provided more evidence to clarify the relationship between air pollution and birth outcomes [4, 5].

The fetal period is a critical period in the development of various systems, during which even slight exposure to chemicals might adversely affect morphology and functions of the systems. Birth weight is an important indicator of fetal growth and nutritional status, which is closely associated with a series of adult health outcomes. Previous studies have suggested that lower birth weight was associated with infant mortality, cardiovascular disease, stroke and type 2 diabetes [6–8]. Additionally, some studies also reported that birth weight loss was also associated with lower intelligence quotient, poorer cognitive functioning and poorer school performance during childhood [9, 10]. Birth weight is affected by many factors, such as maternal age, nutrition and smoking. Studies have found that maternal exposure to lower concentrations of pollutants may also affect fetal health, such as PAHs are a kind of endocrine disruptors that can cross the placental barrier into the fetus from the mother [11–13].

PAHs are generally produced during incomplete combustion processes. Over the past few centuries, as the abundant use of fossil fuels for industrial applications, heating, transport, the emissions of PAHs have become more and more serious. Thus, PAHs are ubiquitous pollutants in both the general environment and in certain working environment [14, 15]. As lipophilic compounds, PAHs can easily cross cell membranes by passive diffusion after inhalation, which might cause an adverse impact on fertility. And since the most of PAHs are present in both in ultrafine size mode (<0.12 μm) and accumulation size mode (0.12–2 μm) [14], it can even get through the placental barrier and enter the fetus from the mother, which may increase the risk of congenital heart defects, neural tube defects and cleft lip/plate [11–13, 16].

A number of recent studies have estimated the association between PAHs exposure during pregnancy and birth weight, but the conclusions were inconsistent and controversial [17–20]. Although some studies suggested that maternal exposure to PAHs was not significantly associated with term low birth weight (LBW, birth weight less than 2500g), some studies have found negative impacts of maternal exposures to PAHs on birth weight [21–27]. A nationwide cohort study in Sweden has suggested that maternal occupational exposure to PAHs significantly increased the risk of LBW (OR:1.49, 95%CI: 1.27, 1.75) [28]. Another study found similar results that placental PAHs levels were negatively associated with birth weight (r: -0.223, P = 0.037) [29]. Choi et al. conducted two parallel prospective cohort studies and found that prenatal PAH exposure was significantly associated with reduced birth weight in both Krakow Caucasians (β: -0.02, 95%CI: -0.03372, -0.00628) and New York City (NYC) African Americans (β: -0.055, 95%CI: -0.0942, -0.0158), but not in NYC Dominicans (β: -0.018, 95%CI: -0.00356, 0.03956) [21]. However, to our knowledge, there is no meta-analysis specifically addressing the association of prenatal exposure to PAHs and birth weight.

Therefore, we performed a meta-analysis to assess the effects of prenatal exposure to PAHs on birth weight. Since humans can be exposed to PAHs through the respiratory tract by inhalation of PAH-containing matter (cigarette smoke, vehicle exhaust, PAH contaminated air emitted from certain industries, etc.) and the digestive tract by intake of PAH-containing foodstuffs (fried and charcoal-grilled meat and PAH contaminated vegetables, etc.) [14]. The exposure levels of PAHs were measured by monitoring station or portable personal air monitoring, PAH-DNA adducts in extracted WBC DNA from cord blood and maternal urinary 1-hydroxy pyrene (1-HP) [30, 31]. Therefore, our meta-analysis also simultaneously included all three method of measuring PAHs exposure. Based on this, to further understand the association between PAHs exposure and birth weight, we collected all data presently available and conducted a meta-analysis on the effects of prenatal exposure to airborne PAHs, PAH-DNA adducts in cord blood and 1-HP in maternal urine on birth weight.

## Materials and methods

Our review was registered with PROSPERO (www.crd.york.ac.uk/PROSPERO) under protocol number CRD42019147060 and developed in accordance with the PRISMA Guidelines in the preparation of this manuscript (www.prisma-statement.org).

### PECO question

The research question was determined using the PECO strategy: Population–newborn and their mother; Exposure–airborne PAHs, PAH-DNA adducts in the umbilical cord blood and 1-HP in maternal urine; Comparison–per Natural logarithm (ln) unit (about 2.72 ng/m^3^) incremental increase in airborne PAHs, high versus low PAH-DNA adducts in the umbilical cord blood and per ln unit μg/g creatinine (about 2.72 μg/g Cr) incremental increase in concentration of 1-HP in maternal urine; and Outcome–birth weight. Based on this, the following research question was established: for newborns, what are the incremental effects of per ln unit exposure to airborne PAHs, high level of PAH-DNA adducts in the umbilical cord blood and per ln unit increase of 1-HP in maternal urine during gestation on birth weight.

### Eligibility criteria

To choose the related articles, we first filtered the titles and abstracts of all studies retrieved from databases. Studies were excluded if they did not related to PAHs and birth weight. Then, from the identified studies, articles were concluded if they: 1) were written in English; 2) just included singleton live birth; 3) were cohort, cross-sectional studies or case control study included only human subject; 4) studied maternal exposure to PAHs during pregnancy and in trimester-specific periods; 5) had excluded women with pregnancy complications or adjusted pregnancy complications as confounding factors; 6) presented the sample sizes and linear regression coefficient (β) with 95% confidence intervals (CIs). In addition, some studies with following criteria were excluded: 1) included premature birth (< 37 weeks) and post-term birth (> 42 weeks) but the gestational week was not adjusted. Studies met all of the above selection criteria were listed for inclusion in the review. When more than one study was determined for a given population, only one study including either the most recent population data or the last information, or both, was selected. Urinary 1-HP is associated with genotoxicity and can serve as a biomarker for internal exposure to PAHs, and the PAH-DNA adducts are also suitable biomarkers for effective dose of PAHs [32]. Previous studies on the detection of PAHs exposure mainly adopt three methods, detecting concentrations of PAHs in the air by portable personal air monitor, detecting the concentration of PAH-DNA adducts in the umbilical cord blood by high-performance liquid chromatography (HPLC) and detecting the concentration of metabolites of 1-HP in urine by HPLC categories. Therefore, we combined and analyzed the included articles according to these three methods.

### Information sources

We searched on PUBMED, the Cochrane Central Register of Controlled Trials (the Cochrane Library), the Web of Science and EMBASE.

### Search

We limited our search to papers published in English until May 8, 2020. All related terms were used for retrieval, such as “polycyclic aromatic hydrocarbons”, “PAH*, “birth weight”, “maternal exposure”, “pregnan*”, “gestation”, “conception”, “gravid*”, “maternal-fetal relations”, “pregnancy outcome”, “birth outcomes”, “birth weight”, “infant”, “newborn”. Full details were provided in the supplementary information (S1 File). In addition, we manually searched the references of each included study for obtaining additional publications.

### Study selection

Two authors worked independently and verified the abstracts and titles of the search results. The differences were resolved by discussion or consultation with a third party to reach consensus. All potentially relevant articles were screened for full text. Where there were differences of opinion, the third author who did not evaluate the articles initially would review it to arrive at a final decision among the three authors. Any reasons for rejection were clearly recorded for the studies that met the inclusion criteria.

### Data collection process

Two authors extracted data using standard template of data extraction independently. The regression coefficients, standard errors, t-values, p-values, mean and median birth weight and 95% confidence intervals of PAHs and birth weight were collected.

### Data items

A Microsoft Excel spreadsheet was used to extract information from eligible studies. The following information was extracted: author, year, title, study design, location and ethnicity, study period, sample population, mean or median age and measure of dispersion, exposure measures, birth weight measures, significance of association, confounders, etc.

### Risk of bias of individual studies

The risk of bias for each included study was accessed using a novel risk of bias instructions for assessing exposure to air pollution developed by Lam [33]. Since the instructions for exposure and outcome assessment domain were not fit for our review, prior to assessing risk of bias, we had modified its evaluation standard, instructions of outcome variable, instructions of incomplete outcome data and instructions of potential cofounding. Full details were provided in the supplementary information (S2 File). The instructions assigned each risk of bias domain as “low”, “probably low”, “probably high”, “high” risk of bias, or “not applicable” (risk of bias area not applicable to study) according to specific criteria as described in the risk of bias instruments. Two authors worked independently and assessed the quality of each study, the differences were resolved by discussion or consultation with a third party to reach consensus. In addition, we evaluated the possibility of publication bias by Begg's test and Egger's test.

### Synthesis of results and statistical analysis

Prior to conducting the meta-analysis, coefficients and their confidence intervals for the associations between PAHs exposure and birth weight from included studies were extracted. Under normal distribution assumption, standard errors (SEs) of the coefficients were calculated by coefficients and their CIs, P-value, sample size and the number of regression parameters with R version 3.6.1. When articles did not report exact P-value, the upper limit of the P value was conservatively taken as the estimated P value on the premise of not changing the total effect (e.g. when P<0.05, the estimated P = 0.05, and when P>0.05, the highest value borrowed from more other studies was taken as the estimated P) [34]. In addition, since all the studies included in our meta-analysis used multiple linear regression model, we converted all regression coefficients into standard regression coefficients. And general linear regression model and semi-logarithmic regression model were converted to logarithmic regression model. Subsequently the coefficients and SEs were converted to the same unit prior to conducting meta-analysis. The unit of the concentration of airborne PAHs is ng/m^3^, and the unit of the concentration of PAH-DNA adducts in cord blood is adducts/10^8^ nucleotides, while the unit of the concentration of 1-HP in maternal urine is μg/g Cr. Heterogeneity was quantified by I^2^ statistics. When significant heterogeneity was present (I^2^> 0.5), a D-L random-effects model meta-analysis was performed to merge effect value. Otherwise, a fixed-effect model meta-analysis was performed. Meta-analysis was performed using “metafor” package in R version 3.6.1. Significance level was set at P < 0.05.

## Result

### Search result

**Fig 1:**
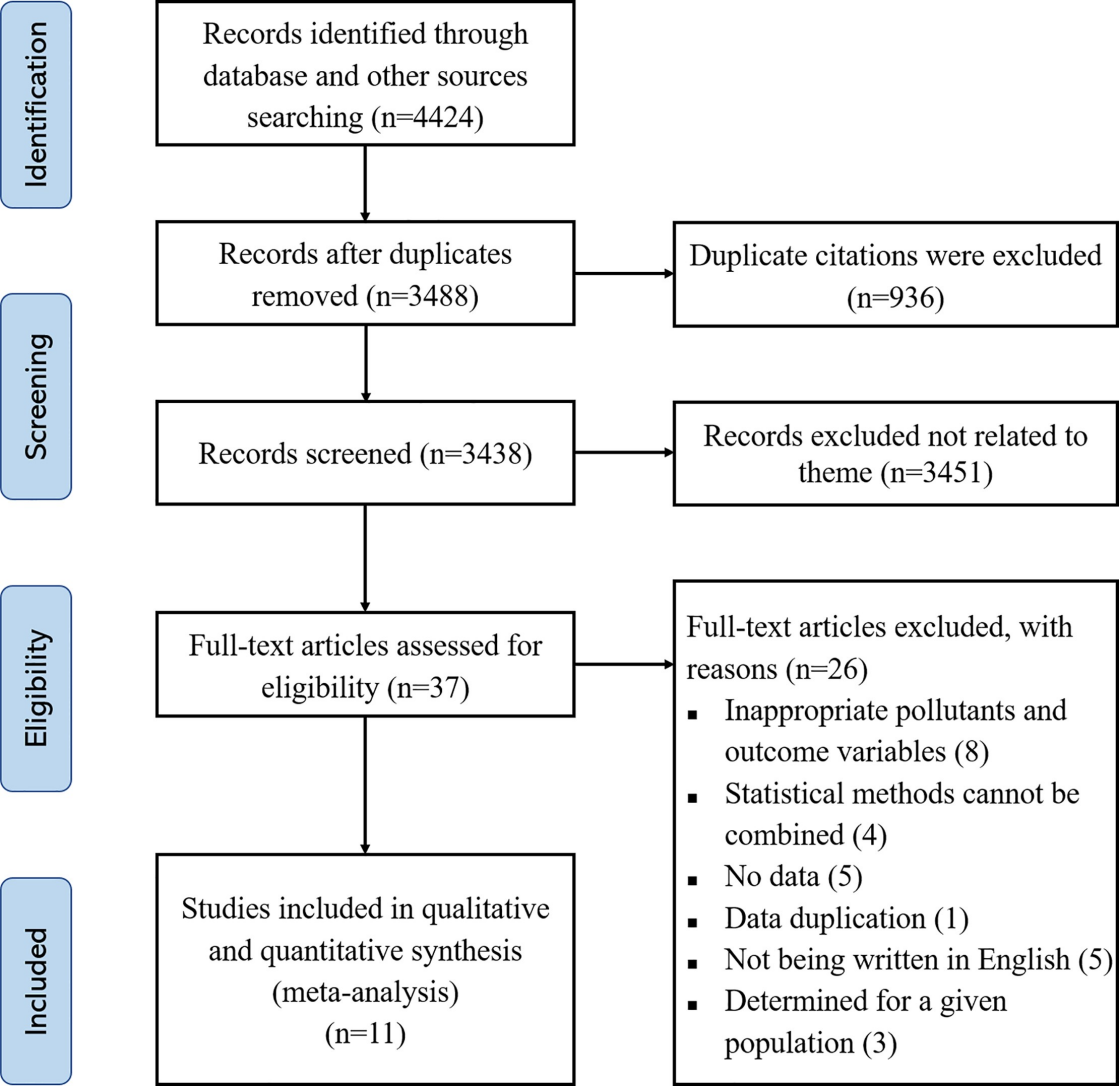
Flow chart of studies selected in meta-analysis.

A total of 3488 citations (excluding duplicates, n = 936) were assessed with the inclusion and exclusion criteria, and after excluding repetition, there were 3,488 remaining. A flowchart of the study selection process was depicted in Fig 1. Finally, a total of 11 studies meeting the inclusion criteria were included in our review, of which 2 citations assessed airborne PAHs exposure, 5 citations assessed PAH-DNA adducts in cord blood, and 4 citations assessed concentration of 1-HP in urine.

### Characteristics of the eligible studies

Table 1 summarized the main parameters from each study. Ten studies were based on prospective cohort study design, and only one was cross-sectional study [19–22, 24–26, 35–38]. The majority of the studies were conducted in the Poland (n = 4), USA (n = 3), followed by China (n = 2), Japan (n = 2) and Saudi Arabia (n = 1). Sample sizes of them ranged from 43 to 1497 participants. Almost all studies adjusted for potential confounders (including maternal education, pre-pregnancy weight, weight gain during pregnancy, sex of infant, gestational age, season of birth and environmental tobacco smoke [ETS]).

**Table 1:** Characteristics of the studies included in the meta-analysis.

Author (Year)	Location (Ethnicity)	Study period	Study design	Data source	Exposure measurement	Sample	Sampling time	β (95%cl)	Exposure Range (Median (IQR)/ Mean ± SD)
Jedrychowski (2017) [22]	Poland (Caucasian)	2000–2004	Prospective birth cohort study	Interview and questionnaire	Portable personal air monitor	455	Second trimester	-0.21(-0.33434, -0.08566)	19.0(47) (ng/m^3^)
Choi (2006) [21]	Poland (Caucasian)	2000–2003	Prospective cohort studies	Interview and questionnaire	Portable personal air monitor	340	Third trimester	-0.02(-0.03372, -0.00628)	39.05±47.63 (ng/m^3^)
Choi (2006) [21]	USA (African Americans)	2000–2003	Prospective cohort studies	Interview and questionnaire	Portable personal air monitor	168	Third trimester	-0.055(-0.0942, -0.0158)	3.34±2.92 (ng/m^3^)
	USA (Dominicans)					212	Third trimester	0.018(-0.00356, 0.03956)	3.72±3.90 (ng/m^3^)
Perera (2004) [23]	USA (African Americans & Dominicans)	No data	Parent cohort of pregnant women and newborns	Interview and questionnaire	Umbilical cord blood	261	Third trimester	-0.02(0.0002, 0.0398)	0.22±0.14 (adducts/10^8^ nucleotides)
Perera (1998) [24]	Poland (Caucasian)	1992	Polish women and newborns cohort	Questionnaire and medical record	Umbilical cord blood	135	Third trimester	-0.044(-0.0873, -0.00035)	0.216±0.856 (adducts/10^8^ nucleotides)
Perera (2005) [20]	USA (No data)	2001–2002	Prospective cohort	Interview and medical record	Umbilical cord blood	181	Third trimester	0.03(-0.0137,0.0737)	0.23±0.10 (adducts/10^8^ nucleotides)
Tang (2014) [25]	China (Chinese)	2005	Prospective cohort	Interview and questionnaire	Umbilical cord blood	158	Third trimester	-0.015(-0.06, 0.03)	0.20±0.08 (adducts/10^8^ nucleotides)
Tang (2006) [26]	China (Chinese)	2002	Prospective cohort	Interview and questionnaire	Umbilical cord blood	150	Third trimester	-0.007(-0.049, 0.035)	0.33±0.14 (adducts/10^8^ nucleotides)
Suzuki (2010) [38]	Japan (Japanese)	2005–2008	Prospective cohort	Medical record	(1-HP) concentration in maternal urine	114	9-40weeks	-0.063(-0.2333, 0.01073)	0.124±0.584 (μg /g Cr)
Niwa (2010) [36]	Japan (Japanese)	2005–2006	Prospective cohort	Medical record	(1-HP) concentration in maternal urine	43	25-38weeks	0.032(-0.054, 0.118)	0.089±3.281 (μg /g Cr)
Polanska (2014) [37]	Poland (Caucasian)	2007–2011	Cohort Study	Questionnaire	(1-HP) concentration in maternal urine	104	20-24weeks	-0.022(-0.0604, 0.0157)	0.430±0.300 (μg /g Cr)
Al-Saleh (2013) [19]	Saudi Arabia (No data)	2005–2006	Cross-sectional study	Questionnaire and clinical file	(1-HP) concentration in maternal urine	1497	Third trimester	0.117(-0.1535, 0.3875)	0.216±0.856 (μg /g Cr)

### Risk of bias

The results showed that these studies had moderate quality. The risk of bias ratings for included studies showed a “PROBABLY HIGH risk of bias” about potential confounding in PAH-DNA adducts of cord blood and 1-HP concentration of maternal urine [25, 36, 38]. Furthermore, one study about PAH-DNA adducts in cord blood was considered as “PROBABLY HIGH” risk of bias for incomplete outcome data and two studies about concentration of 1-HP in maternal urine were considered as “PROBABLY HIGH” risk of bias for participants [24, 36, 38]. Other aspects were generally considered as “LOW” or “PROBABLY LOW” risk of bias. And we evaluated a publication bias among the three groups with Begg's test and Egger's test, only “exposure to airborne PAHs” group had a publication bias found by Egger's test (P = 0.006) (Table 2)

**Table 2:** Pooled association between PAHs exposure and birth weight in different subgroups.

Groups	P (estimate)	OR (95%CI)	P (heterogeneity)	I^2^ (%)	P (Egger's)	P (Begg's)
Group A	0.1131	0.97 (0.93,1.01)	<0.0001	87.17	0.0060*	0.3333
Group B	0.9805	1.00 (0.97,1.03)	0.0533	57.13	0.2911	0.4833
Group C	0.8874	1.00 (0.97,1.03)	0.5864	0.00	0.5920	0.7500

*mean that the result is statistically significant.

Group A: the association between airborne PAHs (per ln unit ng/m^3^ increase) and term birth weight; Group B: the association between PAH-DNA adducts in cord blood (low/high) and term birth weight; Group C: the association between (1-HP) concentration in maternal urine (per ln unit μg/g Cr increase) and term birth weight.

### Qualitative data synthesis

#### Exposure to airborne PAHs

**Fig 2:**
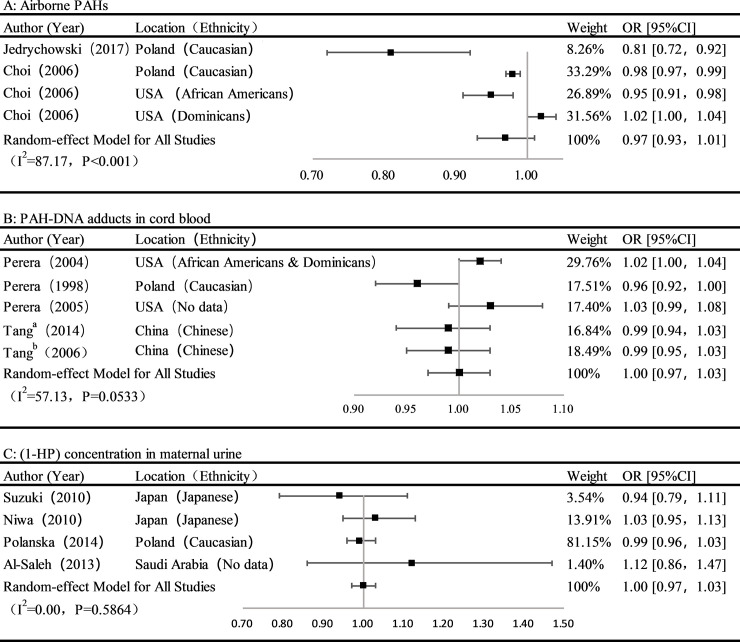
Forest plot showing the risks of PAHs expose and birth weight reduction during the pregnancy period. A: The association between airborne PAHs (per ln unit ng/m^3^ increase) and term birth weight; B: The association between PAH-DNA adducts in cord blood (high vs low) and term birth weight; C: The association between 1-HP concentration in maternal urine (per ln unit μg/g Cr increase) and term birth weight. a: In the cohort, the subjects gave birth between 4 March 2002 and 19 June 2002; b: In the cohort, the subjects gave birth from 2 March 2005 to 23 May 2005.

**Fig 3:**
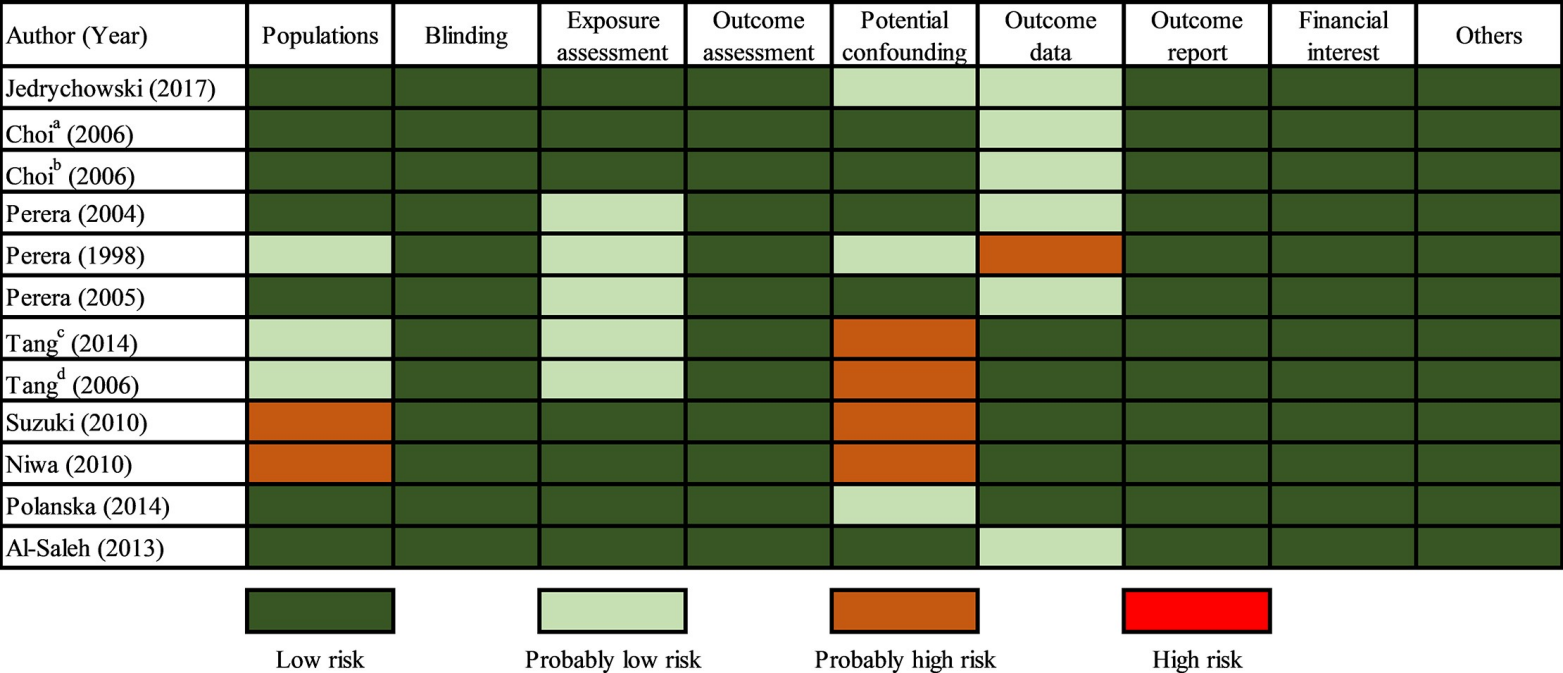
Risk of bias ratings for included studies. ^a^: The participants were from Poland, ^b^: The participants were from New York; ^c, d^: The participants were from different populations.

Two articles [21, 22] were incorporated into the meta-analysis about the association between airborne PAHs exposure and the risk of birth weight reduction. Overall, it showed that per ln unit ng/m^3^ increase of airborne PAHs was not significantly associated with birth weight (OR: 0.97, 95%Cl, 0.93–1.01), with a high heterogeneity (I^2^ = 87.17%) (Fig 2A). And ethnicity (African American, Caucasian, Dominican) and significant differences in PAHs exposure levels might be the main factors leading to high heterogeneity. Although there was a high loss of follow-up rate and incomplete outcome data rate in studies, no significant differences were found between the missing and including subjects in each study in terms of sociodemographic characteristics or exposure levels. The risk of bias ratings for all included studies showed there was a “PROBABLY LOW” risk of bias about incomplete outcome data. (Fig 3)

#### PAH-DNA adducts in cord blood

Fig 2B showed the estimated effect of PAH-DNA adducts in cord blood exposure on birth weight from five studies. Pooled estimates suggested that, comparing with low PAH-DNA adducts, high PAH-DNA adducts exposure did not significantly increase the risk of birth weight reduction (OR: 1.0, 95%Cl: 0.97–1.03). And the heterogeneity among studies assessing PAH-DNA adducts in umbilical cord blood was moderate (I^2^ = 57.13%) (Fig 2B). A study was considered as “PROBABLY HIGH” risk of bias for incomplete outcome data, since it did not explain the reasons and further analysis of incomplete outcome data. Two studies were considered as “PROBABLY HIGH” risk of bias since potential confounding factors were not fully considered. (Fig 3)

#### Concentration of 1-HP in maternal urine

Fig 2C showed the estimated effect of 1-HP concentration in maternal urine on birth weight. Five studies were incorporated into the meta-analysis of the effect of per ln unit μg/g Cr (about 2.72 μg/g Cr) increase of 1-HP in maternal urine on the risk of birth weight reduction. The results of the meta-analysis showed that the association between 1-HP concentration in maternal urine and birth weight was not significant (OR: 1.00, 95%Cl: 0.97–1.03). The heterogeneity of assessing 1-HP concentration in maternal urine (I^2^ = 0.00%) studies was very low. Two studies gave only brief descriptions of the participants, so they were considered as “PROBABLY HIGH” risk of bias. Potential confounding of two studies were considered as “PROBABLY HIGH” risk of bias for lack of important covariates (ETS). (Fig 3)

## Discussion

### Pooled estimates

In this meta-analysis, we provided a summary of the current scientific evidence, and evaluated the overall association between prenatal exposure to PAHs and birth weight. We found that there were no associations between prenatal maternal exposures to airborne PAHs, PAH-DNA adducts in cord blood (low/high) and 1-HP concentration in maternal urine and birth weight. We identified 11 previous researches that link PAHs to birth weight. The regression coefficient of birth weight ranged from -0.21 to 0.117 across the studies. As a whole, most studies were rated as “Low” or “Probably Low” risk of bias in most domains, but one third of the studies had a “Probably High” risk of bias in potential confounding.

The pooled OR for the effect of maternal exposure to airborne PAHs (per ln unit ng/m^3^ increase) during gestation on term birth weight revealed a non-significant positive role (OR: 0.97, 95%CI: 0.93–1.01). This pooled result was consistent with one included study conducted in the NYC Dominican, but the other three included studies all had suggested significantly effects of maternal exposure to airborne PAHs on birth weight [21–23]. It may be due to the large sample size of the former, which leaded to a large weight, thus affecting the overall results. Most of the studies were conducted in the USA and Poland, but the results were not consistent. Choi et al. reported per ln unit ng/m^3^ increase of prenatal PAHs exposure was associated with a 2-fold increase in risk of symmetric intrauterine growth restriction among full-term African American newborns, but this effect was not observed in Dominicans [39]. Dejmek et al. also proved that exposure to PAHs in early gestation may influence fetal growth [40]. Veleminsky et al. suggested that higher PAHs exposure might induce genetic damage in newborns and deregulate genes for immunity in umbilical cord blood, which can increase the risk of intrauterine growth retardation (IUGR) and LBW in newborns [41].

Our meta-analysis suggested that PAH-DNA adducts in cord blood was not significantly associated with term birth weight, which is consistent with Maciel-Ruiz et al. [42]. PAHs, as endocrine disruptors, have antiestrogenic effects, which can bind to the human aryl hydrocarbon receptor to induce P450 enzymes, then DNA damage resulting in activation of apoptotic pathways, finally bind to receptors for placental growth factors resulting in decreased exchange of oxygen and nutrients [25, 43]. Several studies observed PAH–DNA adducts, in conjunction with ETS exposure, were significantly associated with reduced birth weight and head circumference [20, 35]. However, after adjusting ETS, the result were not statistically significant.

In this study, we found no significant association between 1-HP concentration in maternal urine and birth weight, which was consistent with the result of Radmacher et al. [44]. Although Polanska’s study was heavily weighted in this group, excluding it did not significantly change the pooled result. But another study conducted in China showed that the levels of four PAHs metabolites in urine were significantly associated with lower neonatal birth weight [18]. As a biomarker of PAHs exposure, 1-HP has gained a strong position [14]. So in this study, we only estimated the effect of 1-HP on birth weight and found no significant effect. But birth weight might be associated with another particular PAHs metabolite or with a combination of multiple PAHs metabolites. Therefore, further reliable studies on the effects of other PAHs metabolites on birth weight are still needed.

### Interesting results in different races and in high exposure dose areas

No significant effects of PAHs on birth weight were found in our study, but those results should be interpreted with caution. Because we found that results seemed to vary by ethnicity. Specifically, we found that maternal airborne PAHs exposure and PAH-DNA adducts for the Caucasian people (Poland) can cause significant effects on birth weight (Fig 2A & 2B) [22, 24], while exposure to airborne PAHs was significantly associated with birth weight among African-American mothers in New York, but not among Dominican in the same city (Fig 2A) [21]. Those differences among different ethnicity might be caused by interactions between maternal PAHs exposure and genetics on PAH–DNA adducts. The production of these adducts might be determined by mother and newborn genes, which can affect the levels of individual exposure, absorption, metabolic activation, and DNA repair [14, 45].

Subtle genetic differences among different races leads to different levels of DNA adducts under ‘PAH low’ and ‘PAH high’ conditions, which may be related to the effect of gene mutation on DNA damage and repair. Iyer et al. [45, 46] had reported interactions between maternal PAHs exposure and genes on PAH-DNA adducts in an NYC and Polish cohort of non-smoking mothers and newborns. They suggested that there was a greater genetic contribution on the adduct level in both Caucasian individuals and African Americans compared with Dominicans. A study conducted in Shanxi, China, also showed that infants in high PAHs exposure and caring for the mutation genotype had significant birth weight reduction. They suggested that newborn’s XPD polymorphism may play a modificative role in the association between PAHs exposure and birth weight, which indicated that there may be gene-environment interaction in this relationship [17]. At present, there are few studies on the gene-environment interaction between genes and PAHs, therefore, no definite conclusion can be drawn.

In addition, the effect difference between Polish Caucasians and NYC African Americans and Dominicans can be explained by the threshold effect to a certain extent. Polish Caucasians had more than 10-fold higher PAHs exposure risk than NYC African Americans and Dominicans, which is consistent with the higher levels of air pollution in Poland [47]. And a study had confirmed that a significant increase for cord blood PAH-DNA adducts with increasing prenatal exposure to airborne PAHs [48]. PAHs are known inducers of CYP enzymes, including *CYP1A2* and *CYP1B1*, which are related to PAHs metabolism, detoxification and repair. And at higher exposure levels, there may be higher baseline levels of these enzymes for PAHs induction [46]. Only when the concentration of PAHs reaches a certain level, PAHs can induce enough related enzymes and displayed the negative effect on birth weight. And most of the studies were conducted in the USA and Poland, only few studies were conducted in countries with large energy consumption and large population, such as China and India, as well as countries in Europe and the Middle East, so this negative pooled estimates might be influenced by the limited study area. Overall, our meta-analysis suggested that the effects of PAHs exposure on birth weight may vary by ethnicity and exposure dose, which help to identify high-risk groups.

### Limitations and strengths

Several limitations should be addressed. Our review covered limited studies in each group, so the test efficiency of publication bias might be insufficient, and the power of the tests for funnel plot asymmetry might be too low to distinguish chance from real asymmetry [34]. There was a significant heterogeneity in the association between maternal airborne PAHs exposure during the third trimester on term birth weight, which might be due to the effects of variables such as ethnicity, exposure, economic status, or study region. What’s more, we only selected studies written in English, so included participants were mainly from the America and Poland, with a few from China and Japan. But our meta-analysis still had some strengths. This review included a number of high-quality studies. And we seriously assessed the risk of bias of each included study from nine aspects. In addition, we divided the included studies into three groups according to different exposure measurement methods and analyzed the relationship between PAHs and birth weight from those three different angles, which strengthens the credibility of the results.

### Suggestion

Further large cohort studies with sufficient data and reliable exposure data are still required to get a better understanding of the associations between PAHs and birth weight. Meanwhile, subsequent studies should consider more comprehensive confounding factors, including more regional populations and more ethnic participants. Subgroup analysis by race is also necessary, which may help to identify high-risk groups.

## Conclusions

This meta-analysis showed that there was no relationship between maternal PAHs exposure and the risk of term birth weight. Additionally, we have identified potential sources of the heterogeneity, including ethnicity, regions, and exposure levels. We also observed that ethnicity may change the effects of PAHs exposure on birth weight, but the studies were too small to conduct a subgroup analysis. Therefore, future reliable large cohort studies are required for a better understanding of the associations, which might help to identify high-risk groups.

## Supporting information

S1 Checklist PRISMA checklist. (DOC)

S1 File Details of search strategy. (DOCX)

S2 File Risk of bias instructions for assessing exposure to air pollution. (DOCX)

## Abbreviations

PAHs: Polycyclic Aromatic Hydrocarbons
NYC: New York City
SD: standard deviation
1-HP: 1-hydroxy pyrene
HPLC: High performance Liquid Chromatography
CIs: confidence intervals
SEs: Standard errors
ETS: environmental tobacco smoke
IQR: interquartile range
USA: the United States of America
IUGR: intrauterine growth retardation
LBW: low birth weight

## References

[1] Li Z, Yuan X, Fu J, Zhang L, Hong L, Hu L, et al Association of ambient air pollutants and birth weight in Ningbo, 2015–2017. Environmental pollution (Barking, Essex: 1987). 2019;249:629–37. 10.1016/j.envpol.2019.03.076 30933760

[2] Zhu PF, Zhang Y, Ban J, Li TT, Shi XM. [Air pollution and adverse birth outcome in China: a comprehensive review]. Zhonghua liu xing bing xue za zhi = Zhonghua liuxingbingxue zazhi. 2017;38(3):393–9. 10.3760/cma.j.issn.0254-6450.2017.03.024 28329947

[3] Padula AM, Huang H, Baer RJ, August LM, Jankowska MM, Jellife-Pawlowski LL, et al Environmental pollution and social factors as contributors to preterm birth in Fresno County. Environmental health: a global access science source. 2018;17(1):70. 30157858 10.1186/s12940-018-0414-x PMC6114053

[4] Guo LQ, Chen Y, Mi BB, Dang SN, Zhao DD, Liu R, et al Ambient air pollution and adverse birth outcomes: a systematic review and meta-analysis. Journal of Zhejiang University Science B. 2019;20(3):238–52. 10.1631/jzus.B1800122 30829011 PMC6421124

[5] Li X, Huang S, Jiao A, Yang X, Yun J, Wang Y, et al Association between ambient fine particulate matter and preterm birth or term low birth weight: An updated systematic review and meta-analysis. Environmental pollution (Barking, Essex: 1987). 2017;227:596–605. 10.1016/j.envpol.2017.03.055 28457735

[6] Shenkin SD, Starr JM, Deary IJ. Birth weight and cognitive ability in childhood: a systematic review. Psychological bulletin. 2004;130(6):989–1013. 10.1037/0033-2909.130.6.989 15535745

[7] Lau C, Rogers JM. Embryonic and fetal programming of physiological disorders in adulthood. Birth defects research Part C, Embryo today: reviews. 2004;72(4):300–12. 10.1002/bdrc.20029 15662709

[8] Zeng P, Zhou X. Causal Association Between Birth Weight and Adult Diseases: Evidence From a Mendelian Randomization Analysis. Frontiers in genetics. 2019;10:618 10.3389/fgene.2019.00618 31354785 PMC6635582

[9] Chaikind S, Corman H. The impact of low birthweight on special education costs. Journal of health economics. 1991;10(3):291–311. 10.1016/0167-6296(91)90031-h 10170854

[10] Matte TD, Bresnahan M, Begg MD, Susser E. Influence of variation in birth weight within normal range and within sibships on IQ at age 7 years: cohort study. BMJ (Clinical research ed). 2001;323(7308):310–4. 10.1136/bmj.323.7308.310 PMC37317 11498487

[11] Perera F, Tang D, Whyatt R, Lederman SA, Jedrychowski W. DNA damage from polycyclic aromatic hydrocarbons measured by benzo[a]pyrene-DNA adducts in mothers and newborns from Northern Manhattan, the World Trade Center Area, Poland, and China. Cancer epidemiology, biomarkers & prevention: a publication of the American Association for Cancer Research, cosponsored by the American Society of Preventive Oncology. 2005;14(3):709–14. 10.1158/1055-9965.EPI-04-0457 15767354

[12] Wang Y, Tian Z, Zhu H, Cheng Z, Kang M, Luo C, et al Polycyclic aromatic hydrocarbons (PAHs) in soils and vegetation near an e-waste recycling site in South China: concentration, distribution, source, and risk assessment. The Science of the total environment. 2012;439:187–93. 10.1016/j.scitotenv.2012.08.018 23063924

[13] Carre J, Gatimel N, Moreau J, Parinaud J, Leandri R. Does air pollution play a role in infertility?: a systematic review. Environmental health: a global access science source. 2017;16(1):82. 28754128 10.1186/s12940-017-0291-8 PMC5534122

[14] Bostrom CE, Gerde P, Hanberg A, Jernstrom B, Johansson C, Kyrklund T, et al Cancer risk assessment, indicators, and guidelines for polycyclic aromatic hydrocarbons in the ambient air. Environ Health Perspect. 2002;110 Suppl 3:451–88. 12060843 10.1289/ehp.110-1241197 PMC1241197

[15] Cathey A, Ferguson KK, McElrath TF, Cantonwine DE, Pace G, Alshawabkeh A, et al Distribution and predictors of urinary polycyclic aromatic hydrocarbon metabolites in two pregnancy cohort studies. Environmental pollution (Barking, Essex: 1987). 2018;232:556–62. 10.1016/j.envpol.2017.09.087 PMC5650937 28993025

[16] Lin SS, Huang Y, Wang CY, Ren AG. [Polycyclic aromatic hydrocarbons exposure and birth defects]. Zhonghua yu fang yi xue za zhi [Chinese journal of preventive medicine]. 2016;50(6):563–8. 10.3760/cma.j.issn.0253-9624.2016.06.019 27256742

[17] Chen C, Cheng Y, Liu J, Li Y. Association between PAHs exposure, genetic polymorphisms of XPD 751 and newborn birth weight. Journal of Environment and Health. 2013;30(3):189–92.

[18] Cheng L, Li Y, Deng Y, Zhang H, Nie J, Niu Q. Effects of maternal prenatal polycyclic aromatic hydrocarbons exposure on physical development of newborns. Chinese Journal of Environmental & Occupational Medicine. 2017;34(5):385–91.

[19] Al-Saleh I, Alsabbahen A, Shinwari N, Billedo G, Mashhour A, Al-Sarraj Y, et al Polycyclic aromatic hydrocarbons (PAHs) as determinants of various anthropometric measures of birth outcome. The Science of the total environment. 2013;444:565–78. 10.1016/j.scitotenv.2012.12.021 23314068

[20] Perera FP, Tang D, Rauh V, Lester K, Tsai WY, Tu YH, et al Relationships among polycyclic aromatic hydrocarbon-DNA adducts, proximity to the World Trade Center, and effects on fetal growth. Environ Health Perspect. 2005;113(8):1062–7. 10.1289/ehp.7908 16079080 PMC1280350

[21] Choi H, Jedrychowski W, Spengler J, Camann DE, Whyatt RM, Rauh V, et al International studies of prenatal exposure to polycyclic aromatic hydrocarbons and fetal growth. Environmental Health Perspectives. 2006;114(11):1744–50. 10.1289/ehp.8982 17107862 PMC1665416

[22] Jedrychowski WA, Majewska R, Spengler JD, Camann D, Roen EL, Perera FP. Prenatal exposure to fine particles and polycyclic aromatic hydrocarbons and birth outcomes: a two-pollutant approach. Int Arch Occup Environ Health. 2017;90(3):255–64. 10.1007/s00420-016-1192-9 28168423 PMC5360842

[23] Perera FP, Rauh V, Tsai WY, Kinney P, Camann D, Barr D, et al Effects of transplacental exposure to environmental pollutants on birth outcomes in a multiethnic population. Environ Health Perspect. 2003;111(2):201–5. 10.1289/ehp.5742 12573906 PMC1241351

[24] Perera FP, Whyatt RM, Jedrychowski W, Rauh V, Manchester D, Santella RM, et al Recent developments in molecular epidemiology: A study of the effects of environmental polycyclic aromatic hydrocarbons on birth outcomes in Poland. Am J Epidemiol. 1998;147(3):309–14. 10.1093/oxfordjournals.aje.a009451 9482506

[25] Tang D, Li TY, Chow JC, Kulkarni SU, Watson JG, Ho SSH, et al Air pollution effects on fetal and child development: A cohort comparison in China. Environmental Pollution. 2014;185:90–6. 10.1016/j.envpol.2013.10.019 24239591

[26] Tang D, Li TY, Liu JJ, Chen YH, Qu L, Perera F. PAH-DNA adducts in cord blood and fetal and child development in a Chinese cohort. Environ Health Perspect. 2006;114(8):1297–300. 10.1289/ehp.8939 16882543 PMC1552014

[27] Huo X, Wu Y, Xu L, Zeng X, Qin Q, Xu X. Maternal urinary metabolites of PAHs and its association with adverse birth outcomes in an intensive e-waste recycling area. Environmental pollution (Barking, Essex: 1987). 2019;245:453–61. 10.1016/j.envpol.2018.10.098 30458375

[28] Norlén F, Gustavsson P, Wiebert P, Rylander L, Westgren M, Plato N, et al Occupational exposure to organic particles and combustion products during pregnancy and birth outcome in a nationwide cohort study in Sweden. Occup Environ Med. 2019;76(8):537–44. 10.1136/oemed-2018-105672 31123077 PMC6703147

[29] Suter MA, Aagaard KM, Coarfa C, Robertson M, Zhou G, Jackson BP, et al Association between elevated placental polycyclic aromatic hydrocarbons (PAHs) and PAH-DNA adducts from Superfund sites in Harris County, and increased risk of preterm birth (PTB). Biochem Biophys Res Commun. 2019;516(2):344–9. 10.1016/j.bbrc.2019.06.049 31208719 PMC6637943

[30] Alexandrov K, Rojas M, Geneste O, Castegnaro M, Camus AM, Petruzzelli S, et al An improved fluorometric assay for dosimetry of benzo(a)pyrene diol-epoxide-DNA adducts in smokers' lung: comparisons with total bulky adducts and aryl hydrocarbon hydroxylase activity. Cancer Res. 1992;52(22):6248–53. 1423269

[31] Al-Saleh I, Arif J, El-Doush I, Al-Sanea N, Jabbar AA, Billedo G, et al Carcinogen DNA adducts and the risk of colon cancer: case-control study. Biomarkers. 2008;13(2):201–16. 10.1080/13547500701775449 18270871

[32] Talaska G, Thoroman J, Schuman B, Kafferlein HU. Biomarkers of polycyclic aromatic hydrocarbon exposure in European coke oven workers. Toxicol Lett. 2014;231(2):213–6. 10.1016/j.toxlet.2014.10.025 25445007

[33] Lam J, Sutton P, Kalkbrenner A, Windham G, Halladay A, Koustas E, et al A Systematic Review and Meta-Analysis of Multiple Airborne Pollutants and Autism Spectrum Disorder. PLoS One. 2016;11(9):e0161851 10.1371/journal.pone.0161851 27653281 PMC5031428

[34] Higgins JPT, Green S. Cochrane Handbook for Systematic Reviews of Interventions: Cochrane Book Series 2008.

[35] Perera FP, Rauh V, Whyatt RM, Tsai WY, Bernert JT, Tu YH, et al Molecular evidence of an interaction between prenatal environmental exposures and birth outcomes in a multiethnic population. Environmental Health Perspectives. 2004;112(5):626–30. 10.1289/ehp.6617 15064172 PMC1241932

[36] Niwa M, Suzuki Y, Yoshinaga J, Watanabe C, Mizumoto Y. Prenatal Exposure to Polycyclic Aromatic Hydrocarbons and Birth Outcomes. Polycyclic Aromatic Compounds. 2011;31(1):16–27.

[37] Polanska K, Dettbarn G, Jurewicz J, Sobala W, Magnus P, Seidel A, et al Effect of prenatal polycyclic aromatic hydrocarbons exposure on birth outcomes: The polish mother and child cohort study. BioMed Research International. 2014;2014. 10.1155/2014/408939 PMC4129920 25140312

[38] Suzuki Y, Niwa M, Yoshinaga J, Mizumoto Y, Serizawa S, Shiraishi H. Prenatal exposure to phthalate esters and PAHs and birth outcomes. Environ Int. 2010;36(7):699–704. 10.1016/j.envint.2010.05.003 20605637

[39] Choi H, Rauh V, Garfinkel R, Tu Y, Perera FP. Prenatal exposure to airborne polycyclic aromatic hydrocarbons and risk of intrauterine growth restriction. Environ Health Perspect. 2008;116(5):658–65. 10.1289/ehp.10958 18470316 PMC2367680

[40] Dejmek J, Solansky I, Benes I, Lenicek J, Sram RJ. The impact of polycyclic aromatic hydrocarbons and fine particles on pregnancy outcome. Environ Health Perspect. 2000;108(12):1159–64. 10.1289/ehp.001081159 11133396 PMC1240197

[41] Veleminsky M Jr., Hanzl M, Sram RJ. The impact of air pollution in the Southern Bohemia Region on fetuses and newborns. Neuro endocrinology letters. 2016;37(suppl 2):52–7. 28233961

[42] Maciel-Ruiz JA, Lopez-Rivera C, Robles-Morales R, Veloz-Martinez MG, Lopez-Arellano R, Rodriguez-Patino G, et al Prenatal exposure to particulate matter and ozone: Bulky DNA adducts, plasma isoprostanes, allele risk variants, and neonate susceptibility in the Mexico City Metropolitan Area. Environ Mol Mutagen. 2019;60(5):428–42. 10.1002/em.22276 30706525

[43] Moorthy B, Chu C, Carlin DJ. Polycyclic aromatic hydrocarbons: from metabolism to lung cancer. Toxicological sciences: an official journal of the Society of Toxicology. 2015;145(1):5–15. 25911656 10.1093/toxsci/kfv040 PMC4408964

[44] Radmacher PG, Looney SW, Myers SR. Polycyclic Aromatic Hydrocarbons in Maternal and Cord Blood Plasma. Polycyclic Aromatic Compounds. 2010;30(3):113–28.

[45] Iyer S, Wang Y, Xiong W, Tang D, Jedrychowski W, Chanock S, et al Significant interactions between maternal PAH exposure and single nucleotide polymorphisms in candidate genes on B [a] P-DNA adducts in a cohort of non-smoking Polish mothers and newborns. Carcinogenesis. 2016;37(11):1110–5. 10.1093/carcin/bgw090 27565807 PMC6402389

[46] Iyer S, Perera F, Zhang B, Chanock S, Wang S, Tang D. Significant interactions between maternal PAH exposure and haplotypes in candidate genes on B[a]P-DNA adducts in a NYC cohort of non-smoking African-American and Dominican mothers and newborns. Carcinogenesis. 2014;35(1):69–75. 10.1093/carcin/bgt339 24177223 PMC3871941

[47] Wang S, Chanock S, Tang D, Li Z, Jedrychowski W, Perera FP. Assessment of interactions between PAH exposure and genetic polymorphisms on PAH-DNA adducts in African American, Dominican, and Caucasian mothers and newborns. Cancer epidemiology, biomarkers & prevention: a publication of the American Association for Cancer Research, cosponsored by the American Society of Preventive Oncology. 2008;17(2):405–13. 10.1158/1055-9965.EPI-07-0695 PMC3171162 18268125

[48] Jedrychowski WA, Perera FP, Tang D, Rauh V, Majewska R, Mroz E, et al The relationship between prenatal exposure to airborne polycyclic aromatic hydrocarbons (PAHs) and PAH-DNA adducts in cord blood. Journal of exposure science & environmental epidemiology. 2013;23(4):371–7. 23299301 10.1038/jes.2012.117 PMC3733112

