# Supplementary material for: The association between prenatal exposure to polycyclic aromatic hydrocarbons and birth weight: A meta-analysis
Source: PLoS One. 2020 Aug 13;15(8):e0236708. doi: 10.1371/journal.pone.0236708 (PMC7425945; doi:10.1371/journal.pone.0236708)
Supplement: S1 File — (DOCX) [file pone.0236708.s002.docx]

Related articles written in English and published before June 18,2019.

The following electronic databases will be searched:

 PubMed

 Web of Science

 Embase

Cochrane Central Register of Controlled Trials

the keywords that will be used in the search strategies:

#1：((Polycyclic Aromatic Hydrocarbons) OR PAH*)

#2：((((((maternal exposure) OR pregnan*) OR gestation) OR conception) OR gravid*) OR Maternal-Fetal Relations)

#3：(((((pregnancy outcome) OR birth outcomes) OR birth weight) OR infant) OR newborn)

#1 AND #2 AND #3

The specific search strategies of each database are as follows:

**PubMed strategy:**

(Polycyclic Aromatic Hydrocarbons [MeSH Terms] OR Polycyclic Aromatic Hydrocarbons [All Fields]) OR PAH*[All Fields]

AND

(maternal exposure [MeSH Terms] OR maternal exposure [All Fields]) OR (Pregnancy [MeSH Terms] OR pregnan* [All Fields]) OR gestation [All Fields] OR conception [All Fields] OR gravid* [All Fields] OR (Maternal-Fetal Relations [MeSH Terms] OR Maternal-Fetal Relations [All Fields])

AND

(pregnancy outcome [MeSH Terms] OR pregnancy outcome [All Fields]) OR birth outcomes [All Fields] OR (birth weight [MeSH Terms] OR birth weight [All Fields]) OR (infant [MeSH Terms] OR infant [All Fields]) OR (newborn [MeSH Terms] OR newborn [All Fields])

**Embase:**

(‘Polycyclic Aromatic Hydrocarbons’/exp) OR PAH*

AND

(‘maternal exposure’/exp OR ‘pregnancy’/exp OR ‘Maternal-Fetal Relations’/exp) OR (maternal AND exposure) OR pregnancy OR (Maternal-Fetal AND Relations) OR pregnan* OR gestation OR conception OR gravid*

AND

(‘pregnancy outcome’/exp OR ‘birth weight’/exp OR ‘infant’/exp OR ‘newborn’/exp) OR birth outcomes OR (pregnancy AND outcome) OR (birth AND weight) OR infant OR newborn

**Web of Science：**

TS= (“Polycyclic Aromatic Hydrocarbons” OR PAH*)

AND

TS= (“maternal exposure” OR pregnan* OR gestation OR conception OR gravid* OR “Maternal-Fetal Relations”)

AND

TS= (“pregnancy outcome” OR birth outcomes OR “birth weight” OR infant OR newborn)

**Cochrane Central Register of Controlled Trials:**

“Polycyclic Aromatic Hydrocarbons” OR PAH*[All Text]

AND

“maternal exposure” OR pregnan* OR gestation OR conception OR gravid* OR “Maternal-Fetal Relations”[All Text]

AND

“pregnancy outcome” OR (birth AND outcomes) OR “birth weight” OR infant OR newborn[All Text]
