# Supplementary material for: The association between prenatal exposure to polycyclic aromatic hydrocarbons and birth weight: A meta-analysis
Source: PLoS One. 2020 Aug 13;15(8):e0236708. doi: 10.1371/journal.pone.0236708 (PMC7425945; doi:10.1371/journal.pone.0236708)
Supplement: S2 File — (DOCX) [file pone.0236708.s003.docx]

**Appendix** Ⅱ**. Instructions for making risk of bias determinations.**

Human Studies

*Please answer LOW RISK, PROBABLY LOW RISK, PROBABLY HIGH RISK, HIGH RISK or NOT APPLICABLE and provide details/justification.*

*Note: These criteria for judging risk of bias are for human studies only since we are not evaluating animal studies in this case study. These questions have also been modified from previous applications of the Navigation Guide, with edits intended so that answering “Yes” to each question aligns with a rating of “High risk of bias”, “Probably Yes” 🡪 “Probably high risk of bias”, “Probably No” 🡪 “Probably low risk of bias” and “No” 🡪 “Low risk of bias.”*

**1. Are the study groups at risk of not representing their source populations in a manner that might introduce selection bias?**

Criteria for a judgment of LOW risk of bias (i.e., answer: “No”):

EITHER:

a) The descriptions of the source population, inclusion/exclusion criteria, recruitment and enrollment procedures, participation and follow-up rates were sufficiently detailed and adequate data on the distribution of relevant study sample and population characteristics were supplied to support the assertion that risk of selection effects was minimal.

OR

b) Although the descriptions and/or data as indicated in “a” above suggested the potential for selection effects, adequate support was given indicating that potential selection effects were *not* differential across both exposure and outcome.

OR

c) Although the descriptions and/or data as indicated in “a” above suggested the potential for selection effects and there was no support indicating that potential selection effects were *not* differential across both exposure and outcome, selection factors appeared to be well-understood, were measured in the data set, and appropriate adjustment post hoc techniques were used to control for selection bias.

Criteria for the judgment of PROBABLY LOW risk of bias (i.e., answer: “Probably No”):

There is insufficient information about participant selection to permit a judgment of low risk of bias, but there is indirect evidence that suggests that inclusion/exclusion criteria, recruitment and enrollment procedures, and participation and follow-up rates were consistent across groups as described by the criteria for a judgment of low risk of bias.

Criteria for the judgment of PROBABLY HIGH risk of bias (i.e., answer: “Probably Yes”):

There is insufficient information about participant selection to permit a judgment of high risk of bias, but there is indirect evidence that suggests that inclusion/exclusion criteria, recruitment and enrollment procedures, and participation and follow-up rates were inconsistent across groups, as described by the criteria for a judgment of high risk of bias.

Criteria for the judgment of HIGH risk of bias (i.e., answer: “Yes”):

1. There were indications from descriptions of the source population, inclusion/exclusion criteria, recruitment and enrollment procedures, participation and follow-up rates and data on the distribution of relevant study sample and population characteristics that risk of selection effects were substantial; and
2. There was no support to indicate that potential selection effects were *not* differential across both exposure and outcome; and
3. Adjustment post hoc techniques were not used to control for selection bias.

Criteria for the judgment of NOT APPLICABLE (risk of bias domain is not applicable to study):

There is evidence that participant selection is not an element of study design capable of introducing risk of bias in the study.

**2. Was knowledge of the group assignments inadequately prevented during the study, potentially leading to subjective measurement of either exposure or outcome?**

Criteria for a judgment of LOW risk of bias (i.e., answer: “No”):

Any of the following:

- No blinding, but the review authors judge that the outcome and the outcome measurement as well as the exposure and exposure measurement are not likely to be influenced by lack of blinding (such as differential outcome assessment where the outcome is assessed using different measurement or estimation metrics across exposure groups, or differential exposure assessment where exposure is assessed using different measurement or estimation metrics across diagnostic or outcome groups); or
- Blinding of key study personnel was ensured, and it is unlikely that the blinding could have been broken; or
- Some key study personnel were not blinded, but exposure and outcome assessment were blinded and the non-blinding of others is unlikely to introduce bias.

Criteria for the judgment of PROBABLY LOW risk of bias (i.e., answer: “Probably No”):

There is insufficient information about blinding to permit a judgment of low risk of bias, but there is indirect evidence that suggests the study was adequately blinded, as described by the criteria for a judgment of low risk of bias. For example, investigators were effectively blinded to the exposure and/or outcome groups, for example if the exposure was measured by a separate entity and the outcome was obtained from a hospital record.

Criteria for the judgment of PROBABLY HIGH risk of bias (i.e., answer: “Probably Yes”):

There is insufficient information about blinding to permit a judgment of high risk of bias, but there is indirect evidence that suggests the study was not adequately blinded, as described by the criteria for a judgment of high risk of bias.

Criteria for the judgment of HIGH risk of bias (i.e., answer: “Yes”):

Any of the following:

- No blinding or incomplete blinding, and the outcome or outcome measurement or exposure and exposure measurement is likely to be influenced by lack of blinding (i.e., differential outcome or exposure assessment); or
- Blinding of key study personnel attempted, but likely that the blinding could have been broken so as to introduce bias; or
- Some key study personnel were not blinded, and the non-blinding of others was likely to introduce bias.

Criteria for the judgment of NOT APPLICABLE (risk of bias domain is not applicable to study):

There is evidence that blinding is not an element of study design capable of introducing risk of bias in the study.

**3. Were exposure assessment methods lacking accuracy, e.g. allowing misclassification?**

*Note: For this risk of bias domain, we will separately consider each exposure assessment metric within the same study since different exposures measures may have different risks of bias, i.e., metals vs. PM, models vs. biomonitoring, etc. We will divide an individual study up into separate data sets according to the number of separate exposures analyzed in the study. For example, if the study categorizes exposures by “organic solvents”, “particulate matter” and “critical air pollutants” we will treat/analyze each of these exposures groups as three separate data sets; if a study assigns an exposure on a chemical by chemical or pollutant by pollutant basis, each chemical will be assessed as a separate data set, etc. Therefore, our review’s denominators will be “X included studies” and “X included data sets”.*

*Risk of bias will be assessed for each data set. The risk of bias over the body of evidence will be rated by review authors’ review of risk of bias across all datasets (not across all studies). Our rationale for breaking up studies into data sets is that: 1) there is empirical evidence that risk of bias varies depending on which air pollution exposure was measured (i.e., chemical component) and how it was measured (i.e., exposure metric) [1]; 2) there is a need to transparently distinguish among these potential biases within a given study; and 3) co-authors in consultation with experts in the field (HC) did not identify an empirically-based or otherwise scientifically preferable alternative method to address this aspect of heterogeneity in the data.*

*The following list of considerations represents a collection of factors proposed by experts in various fields that may potentially influence the internal validity of the exposure assessment in a systematic manner (not those that may randomly affect overall study results).* ***These should be interpreted only as suggested considerations, and should not be viewed as scoring or a checklist.***

**List of Considerations**:

*Exposure assessment metric:*

1. *Modeling*
2. *Monitoring*
3. *Biomarkers*

*For each, overall considerations include:*

1. *What is the quality of the metric being used?*
2. *Has the metric been validated for the scenario for which it is being used?*
3. *Are the pollutants measured in the study primary air pollutants (CO2, NOx, etc.), secondary air pollutants (O3), or neither primary nor secondary air pollutants (metals, pesticides, etc.)?*
4. *Is the exposure measured in the study a surrogate for air pollution (i.e., distance to freeway)?*
5. *What was the temporal coverage (i.e., whole developmental period, or a shorter duration)?*
6. *Did the analysis account for prediction uncertainty?*
7. *How was missing data accounted for, and any data imputations incorporated?*
8. *Were sensitivity analyses performed?*

*In particular, for exposure assessment models:*

1. *Were the input data in the study suspected to systematically under- or over-estimate exposure?*
2. *What type of model was used (geostatistical interpolation, land-use regression, dispersion models, personal air sampling models, hybrid models, etc.)?*
3. *Were meteorological variables incorporated in the model and justified by authors in their selection?*
4. *Were data on land use, topography, traffic, monitoring data, emission rates, etc. incorporated and justified by authors in their selection?*
5. *What was the spatial variation (e.g., distance from source) and geographic/spatial accuracy (county, census tract, individual residence)?*
6. *What was the temporal specificity and variation (accuracy to level of the day, pregnancy trimester, year, etc.?)*
7. *What was the address completeness (e.g., only home address at one point in time, or more complete address history throughout pregnancy/postnatal life and other locations such as work)?*
8. *What was the space-time coverage of the model?*
9. *Were time-activity patterns accounted for?*
10. *Was mixing height considered as a covariate?*

Criteria for a judgment of LOW risk of bias (i.e., answer: “No”):

The reviewers judge that there is low risk of exposure misclassification, i.e.:

- There is high confidence in the accuracy of the exposure assessment methods, such as methods that have been tested for validity and reliability in measuring the targeted exposure; or
- Less-established or less direct exposure measurements are validated against well-established or direct methods; or

1. Biomarkers: a direct measure of two or more constituents of air pollution exposure during the time period that exposure is considered relevant (i.e., developmental period as defined in the PECO statement) was used, and there is sufficient evidence that relevant factors from the List of Considerations above would imply minimal risk of bias in the exposure assessment; or
2. Monitoring: direct and personal monitoring devices that were used that have been validated for the chemical and scenario for which it was used and there is sufficient evidence that relevant factors from the List of Considerations above would imply minimal risk of bias in the exposure assessment; or
3. Modeling: the model accounted for the time-activity pattern specific to each research participant, (e.g. includes more than exposure at the residential address) and included air pollution modeling methods that have been validated or shown to have a high degree of spatial accuracy (e.g. point location), and/or methods that are themselves validated with good agreement compared to person-based air data collection; and there is sufficient evidence that relevant factors from the List of Considerations above would imply minimal risk of bias in the exposure assessment.

AND if applicable (e.g. for laboratory measurements), appropriate QA/QC for methods are described and are satisfactory, with at least three of the following items reported, or at least two of the following items reported plus evidence of satisfactory performance in a high quality inter-laboratory comparison:

- Limit of detection or quantification;
- standards recovery;
- measure of repeatability;
- investigation and prevention of blanks contamination.

Criteria for the judgment of PROBABLY LOW risk of bias (i.e., answer: “Probably No”):

There is insufficient information about the exposure assessment methods to permit a judgment of low risk of bias, but there is indirect evidence that suggests that methods were robust, as described by the criteria for a judgment of low risk of bias. Studies only reporting that the QA/QC items above were satisfactory but not reporting all of the actual numbers may receive a judgment of “probably low risk of bias.” Additionally:

1. Biomarkers: a measure that included at least 1 constituent of air pollution exposure during the time period that exposure is considered relevant and has been validated as a direct measure of exposure (i.e., developmental period as defined in the PECO statement) was used, or there is some evidence that relevant factors from the List of Considerations above would imply minimal risk of bias in the exposure assessment.
2. Monitoring: methodologies which directly assess exposure were used, such as personal exposure instruments, but had not been validated for that purpose, or if such instruments were worn for less than 4 hours per day, or there is some evidence that relevant factors from the List of Considerations above would imply minimal risk of bias in the exposure assessment.
3. Modeling: the model used methods that do not meet the criteria of including time-activity patterns AND spatial accuracy, and so may not have the level of validation compared to person-based air measurement, but include measurements that have evidence of quality, such as good-quality data inputs, validation against area-based air measurement, or other establishments of the accuracy of the data inputs and models, or there is some evidence that relevant factors from the List of Considerations above would imply minimal risk of bias in the exposure assessment.

Criteria for the judgment of PROBABLY HIGH risk of bias (i.e., answer: “Probably Yes”):

There is insufficient information about the exposure assessment methods to permit a judgment of high risk of bias, but there is indirect evidence that suggests that methods were not robust, as described by the criteria for a judgment of high risk of bias. Additionally:

1. Biomarkers: this includes indirect measures of exposure of air pollution but not specific to this exposure, such as DNA adducts, inflammation or oxidative stress, during the time period that exposure is considered relevant (i.e., developmental period as defined in the PECO statement), or there is some evidence that relevant factors from the List of Considerations above would imply risk of bias in the exposure assessment.
2. Monitoring: measurement of exposures that may not have been validated for use to study air pollution were used, or there is some evidence that relevant factors from the List of Considerations above would imply risk of bias in the exposure assessment.
3. Modeling: air pollution models were used that have not been compared to person-based or area-based air measurements and have suspicion of problems estimating true exposure because, for example, they do not have spatial accuracy (e.g. county-level measures), do not pertain to the correct time frame, are based on limited data, or differ in methodology between cases and controls in a study, or there is some evidence that relevant factors from the List of Considerations above would imply risk of bias in the exposure assessment.

Criteria for the judgment of HIGH risk of bias (i.e., answer: “Yes”):

The reviewers judge that there is high risk of exposure misclassification and any one of the following:

- There is low confidence in the accuracy of the exposure assessment methods; or
- Less-established or less direct exposure measurements are not validated and are suspected to introduce bias that impacts the outcome assessment (example: participants are asked to report exposure status retrospectively, subject to recall bias); or
- Uncertain how exposure information was obtained; or:

1. Biomarkers: There is sufficient evidence that relevant factors from the List of Considerations above would imply risk of bias in the exposure assessment.
2. Monitoring: Information from databases or otherwise was gathered that indirectly assessed exposure without considering variables noted in the List of Considerations above, such as spatial variability, land use regression, etc., or there is sufficient evidence that relevant factors from the List of Considerations above would imply risk of bias in the exposure assessment.
3. Modeling: the air pollution model used has been demonstrated not to pertain to area-based or person-based measures or has otherwise been previously demonstrated to be unable to describe air levels of exposure for assigning exposure in a research situation, or there is sufficient evidence that relevant factors from the List of Considerations above would imply risk of bias in the exposure assessment.

Criteria for the judgment of NOT APPLICABLE (risk of bias domain is not applicable to study):

There is evidence that exposure assessment methods are not capable of introducing risk of bias in the study.

A longitudinal study in which researchers used 2 different editions of the same tool that otherwise met the above criteria

**4. Were outcome assessment methods lacking accuracy?**

Criteria for a judgment of LOW risk of bias (i.e., answer: “No”): (i.e. Tier 1)

- The outcome of newborns was measured by clinical nurse at delivery with the standard measurement procedures.
- The information obtained from medical record and clinical file.

Criteria for the judgment of PROBABLY LOW risk of bias (i.e., answer: “Probably No”): (i.e. Tier 2)

Any of the following:

- The outcome of newborns was not measured by clinical nurse at delivery with the standard measurement procedures. And the information obtained from medical record and clinical file; or
- The outcome of newborns was measured by clinical nurse at delivery with the standard measurement procedures. But the information did not obtain from medical record and clinical file but from questionnaire or interviews with mothers.

Criteria for the judgment of PROBABLY HIGH risk of bias (i.e., answer: “Probably Yes”): (Tier 3)

- The outcome of newborns was not measured by clinical nurse at delivery with the standard measurement procedures. And the information did not obtain from medical record and clinical file but from questionnaire or interviews with mothers.

Criteria for the judgment of HIGH risk of bias (i.e., answer: “Yes”):

- None of the actions described above were described

**5. Was potential confounding inadequately incorporated?**

*Single birth and gestational age, the potentially important confounders, were our inclusion and exclusion criteria, we have described this in the methods section. Prior to the evaluation of studies, coauthors collectively developed the following list of potentially important confounders as well as the rationale for inclusion.*

*1.  Social class.*

*This is measured differently from study to study, such as by education, income, race.  Note that variables like marital status and insurance can even reflect aspects of social class.  Sometimes social class is accounted for by individual-level measurements, and other times by group-level measurements (such as census variables).*

*Rationale:  Where people live (neighborhood) is strongly influenced by social class.  And the airborne pollutants that someone is exposed to are influenced by neighborhood, so social class is related to neighborhood. It's possible that a measured link between air pollutants and birth weight could be influenced artificially (confounded) by unknown aspects of social class.*

*2.  Maternal (and/or paternal) age.*

*Maternal age is related to social class, because very young mothers tend to be of lower social class and older mothers tend to be of higher social class so maternal age may be correlated with air pollutant levels (i.e., younger women may be of lower social class, and lower social classes may be exposed to higher levels of air pollutants).  Note that while paternal age is likely also a risk factor, it is often not adjusted for because this variable has a high degree of missing-ness.  Because maternal age and paternal age are highly related, it is often thought that adjusting for maternal age is sufficient, and so may not be necessary to include both.*

*3.  Season of conception/birth (calendar time of conception/birth).*

*Air pollutant concentrations also vary by season due to sunlight and other factors.  Air pollutants will only vary by season if there is temporal refinement in the air pollutant measure, such as monthly or trimester-long values.  A study with annual averages or air pollutant levels, or static levels such as distance to a road, will NOT show a correlation structure between season and air pollutants, and so season will not confound in this type of study.  An observed relationship between air pollutants and autism could be driven by the factors responsible for the seasonal pattern in autism.  Note that it is unknown what these factors are - speculation is about Vitamin D levels, flu season, etc.*

*4.* *Maternal environmental tobacco smoke (ETS) exposure*

*Environmental tobacco smoke is an important source of Polycyclic Aromatic Hydrocarbons exposure. It has been estimated that active smoking in pregnancy leads to a reduction in birth weight*

*, and exposure to environmental tobacco smoke in pregnancy results in birth weight reduction by approximately 20–30 g.[2]*

Criteria for a judgment of LOW risk of bias (i.e., answer: “No”):

The study appropriately assessed and accounted for (i.e., matched, stratified, or statistically controlled for) all important potential confounders, or reported that potential confounders were evaluated and omitted because inclusion did not substantially affect the results. The determination of specific confounders may be informed by, but not limited to, the studies included in the overall review.

AND the important potential confounders were measured consistently across study groups using valid and reliable methods, or the influence of covariate measurement error was determined, through sensitivity analysis, to be minimal.

Criteria for the judgment of PROBABLY LOW risk of bias (i.e., answer: “Probably No”):

The study appropriately accounted for most but not all of the important potential confounders

AND this is not expected to introduce substantial bias.

Criteria for the judgment of PROBABLY HIGH risk of bias (i.e., answer: “Probably Yes”):

The study evaluated some but not all of the important potential confounders

AND this is expected to introduce substantial bias.

Criteria for the judgment of HIGH risk of bias (i.e., answer: “Yes”):

The study did not account for or evaluate multiple important potential confounders.

OR the important potential confounders were inappropriately measured and/or inappropriately analyzed across study groups.

Criteria for the judgment of NOT APPLICABLE (risk of bias domain is not applicable to study):

There is evidence that outcome assessment methods are not capable of introducing risk of bias in the study.

**6. Were** **incomplete outcome data inadequately addressed?**

Criteria for a judgment of LOW risk of bias (i.e., answer: “No”):

Participants were followed long enough to obtain outcome measurements

OR any one of the following:

- No missing outcome data; or
- Reasons for missing outcome data unlikely to be related to true outcome (for survival data, censoring unlikely to introduce bias); or
- Attrition or missing outcome data balanced in numbers across exposure groups, with similar reasons for missing data across groups; or
- For dichotomous outcome data, the proportion of missing outcomes compared with observed event risk not enough to have a relevant impact on the intervention effect estimate; or
- For continuous outcome data, plausible effect size (difference in means or standardized difference in means) among missing outcomes not enough to have a relevant impact on the observed effect size; or
- Missing data have been imputed using appropriate methods.

Criteria for the judgment of PROBABLY LOW risk of bias (i.e., answer: “Probably No”):

There is insufficient information about incomplete outcome data to permit a judgment of low risk of bias, but there is indirect evidence that suggests incomplete outcome data was adequately addressed, as described by the criteria for a judgment of low risk of bias.

Criteria for the judgment of PROBABLY HIGH risk of bias (i.e., answer: “Probably Yes”):

- There is insufficient information about incomplete outcome data to permit a judgment of high risk of bias, but there is indirect evidence that suggests incomplete outcome data was not adequately addressed, as described by the criteria for a judgment of high risk of bias; or there were no significant differences in sociodemographic characteristics or levels of exposure between subjects with missing data and those included in each study.

Criteria for the judgment of HIGH risk of bias (i.e., answer: “Yes”):

Participants were not followed long enough to obtain outcome measurements

OR any one of the following:

- Reason for missing outcome data likely to be related to true outcome, with either imbalance in numbers or reasons for missing data across exposure groups; or
- For dichotomous outcome data, the proportion of missing outcomes compared with observed event risk enough to induce biologically relevant bias in intervention effect estimate; or
- For continuous outcome data, plausible effect size (difference in means or standardized difference in means) among missing outcomes enough to induce biologically relevant bias in observed effect size; or
- Potentially inappropriate application of imputation.

Criteria for the judgment of NOT APPLICABLE (risk of bias domain is not applicable to study):

There is evidence that incomplete outcome data is not capable of introducing risk of bias in the study.

**7.** **Does the study report appear to have selective outcome reporting?**

Criteria for a judgment of LOW risk of bias (i.e., answer: “No”):

All of the study’s pre-specified (primary and secondary) outcomes outlined in the protocol, methods, abstract, and/or introduction that are of interest in the review have been reported in the pre-specified way.

Criteria for the judgment of PROBABLY LOW risk of bias (i.e., answer: “Probably No”):

There is insufficient information about selective outcome reporting to permit a judgment of low risk of bias, but there is indirect evidence that suggests the study was free of selective reporting, as described by the criteria for a judgment of low risk of bias.

Criteria for the judgment of PROBABLY HIGH risk of bias (i.e., answer: “Probably Yes”):

There is insufficient information about selective outcome reporting to permit a judgment of high risk of bias, but there is indirect evidence that suggests the study was not free of selective reporting, as described by the criteria for a judgment of high risk of bias.

Criteria for the judgment of HIGH risk of bias (i.e., answer: “Yes”):

Any one of the following:

- Not all of the study’s pre-specified primary outcomes (as outlined in the protocol, methods, abstract, and/or introduction) have been reported; or
- One or more primary outcomes is reported using measurements, analysis methods or subsets of the data (e.g. subscales) that were not pre-specified; or
- One or more reported primary outcomes were not pre-specified (unless clear justification for their reporting is provided, such as an unexpected effect); or
- One or more outcomes of interest are reported incompletely

Criteria for the judgment of NOT APPLICABLE (risk of bias domain is not applicable to study):

There is evidence that selective outcome reporting is not capable of introducing risk of bias in the study.

**8. Did the study receive any support from a company, study author, or other entity having a financial interest in any of the exposures studied?**

Criteria for a judgment of LOW risk of bias (i.e., answer: “No”):

The study did not receive support from a company, study author, or other entity having a financial interest in the outcome of the study. Examples include the following:

- Funding source is limited to government, non-profit organizations, or academic grants funded by government, foundations and/or non-profit organizations;
- Chemicals or other treatment used in study were purchased from a supplier;
- Company affiliated staff are not mentioned in the acknowledgements section;
- Authors were not employees of a company with a financial interest in the outcome of the study;
- Company with a financial interest in the outcome of the study was not involved in the design, conduct, analysis, or reporting of the study and authors had complete access to the data;
- Study authors make a claim denying conflicts of interest;
- Study authors are unaffiliated with companies with financial interest, and there is no reason to believe a conflict of interest exists;
- All study authors are affiliated with a government agency (are prohibited from involvement in projects for which there is a conflict of interest or an appearance of conflict of interest).

Criteria for the judgment of PROBABLY LOW risk of bias (i.e., answer: “Probably No”):

There is insufficient information to permit a judgment of low risk of bias, but there is indirect evidence that suggests the study was free of support from a company, study author, or other entity having a financial interest in the outcome of the study, as described by the criteria for a judgment of low risk of bias.

Criteria for the judgment of PROBABLY HIGH risk of bias (i.e., answer: “Probably Yes”):

There is insufficient information to permit a judgment of high risk of bias, but there is indirect evidence that suggests the study was not free of support from a company, study author, or other entity having a financial interest in the outcome of the study, as described by the criteria for a judgment of high risk of bias.

Criteria for the judgment of HIGH risk of bias (i.e., answer: “Yes”):

The study received support from a company, study author, or other entity having a financial interest in the outcome of the study. Examples of support include:

- Research funds;
- Chemicals, equipment or testing provided at no cost;
- Writing services;
- Author/staff from study was employee or otherwise affiliated with company with financial interest;
- Company limited author access to the data;
- Company was involved in the design, conduct, analysis, or reporting of the study;
- Study authors claim a conflict of interest

Criteria for the judgment of NOT APPLICABLE (risk of bias domain is not applicable to study):

There is evidence that conflicts of interest are not capable of introducing risk of bias in the study.

**9. Did the study appear to have other problems that could put it at a risk of bias?**

Criteria for a judgment of LOW risk of bias (i.e., answer: “No”):

The study appears to be free of other sources of bias.

Criteria for the judgment of PROBABLY LOW risk of bias (i.e., answer: “Probably No”):

There is insufficient information to permit a judgment of low risk of bias, but there is indirect evidence that suggests the study was free of other threats to validity.

Criteria for the judgment of PROBABLY HIGH risk of bias (i.e., answer: “Probably Yes”):

There is insufficient information to permit a judgment of high risk of bias, but there is indirect evidence that suggests the study was not free of other threats to validity, as described by the criteria for a judgment of high risk of bias.

Criteria for the judgment of HIGH risk of bias (i.e., answer: “Yes”):

There is at least one important risk of bias. For example, the study:

- Had a potential source of bias related to the specific study design used; or
- Stopped early due to some data-dependent process (including a formal-stopping rule); or
- The conduct of the study is affected by interim results (e.g. recruiting additional participants from a subgroup showing greater or lesser effect); or
- Has been claimed to have been fraudulent; or
- Had some other problem

**References**

1. US Environmental Protection Agency. National Air Toxics Assessments 2013 [cited 2015 01-30]. Available from: <http://www.epa.gov/airtoxics/natamain/>.

2. Sram RJ, Binkova B, Dejmek J, Bobak M. Ambient air pollution and pregnancy outcomes: a review of the literature. Environ Health Perspect. 2005;113(4):375-82. Epub 2005/04/07. doi: 10.1289/ehp.6362. PubMed PMID: 15811825; PubMed Central PMCID: PMCPMC1278474.
